# Correction: The impact of long-term care insurance on family care for older adults: The mediating role of intergenerational financial support

**DOI:** 10.1371/journal.pone.0312716

**Published:** 2024-10-23

**Authors:** The PLOS ONE Staff

## Notice of Republication

This article was republished on October 14, 2024, to correct an error in the author list: Lianjie Wang was incorrectly designated as the corresponding author. The publisher apologizes for the error. Please download this article again to view the correct version. The originally published, uncorrected article and the republished, corrected article are provided here for reference.

## Supporting information

S1 File Originally published, uncorrected article. (PDF)

S2 File Republished, corrected article. (PDF)
