# Supplementary material for: The impact of long-term care insurance on family care for older adults: The mediating role of intergenerational financial support
Source: PLoS One. 2024 May 23;19(5):e0299974. doi: 10.1371/journal.pone.0299974 (PMC11115205; doi:10.1371/journal.pone.0299974)
Supplement: Attachment — Submitted filename: Response to Reviewers.docx [file pone.0299974.s001.docx]

**Response to Reviewers**

Dear Editor and Reviewers,

We would like to express our gratitude for your professional suggestions and constructive comments, which are essential for improving the quality of our research paper. We have carefully considered and addressed each point raised by the reviewers, and we believe that our revised manuscript is now significantly improved. Please find below a detailed response to each of the reviewers' comments.

**Journal requirements:**

When submitting your revision, we need you to address these additional requirements.

**1. Please ensure that your manuscript meets PLOS ONE's style requirements, including those for file naming. The PLOS ONE style templates can be found at https://journals.plos.org/plosone/s/file?id=wjVg/PLOSOne_formatting_sample_main_body.pdf https://journals.plos.org/plosone/s/file?id=ba62/PLOSOne_formatting**

**_sample_title_authors_affiliations.pdf.**

**Response:**

Thank you for the suggestions provided by the journal. We have made modifications to the format of this article according to the requirements of the two documents, making the manuscript more in line with the journal’s requirements.

**2. We note that the grant information you provided in the ‘Funding Information’ and ‘Financial Disclosure’ sections do not match. When you resubmit, please ensure that you provide the correct grant numbers for the awards you received for your study in the ‘Funding Information’ section.**

**Response:**

Thank you for your reminder. We have carefully checked and corrected the funding detail.

**3. We note that you have stated that you will provide repository information for your data at acceptance. Should your manuscript be accepted for publication, we will hold it until you provide the relevant accession numbers or DOIs necessary to access your data. If you wish to make changes to your Data Availability statement, please describe these changes in your cover letter and we will update your Data Availability statement to reflect the information you provide.**

**Response:**

Thank you so much for this kind reminder. The data we used in this article is secondary data, which is openly available to the public. If you need anything else from us, we will be more than happy to provide

**4. Please include your full ethics statement in the ‘Methods’ section of your manuscript file. In your statement, please include the full name of the IRB or ethics committee who approved or waived your study, as well as whether or not you obtained informed written or verbal consent. If consent was waived for your study, please include this information in your statement as well.**

**Response:**

Thank you for your comment. We have added the "Ethics statement" in the "Methods" section of this paper on page 11.

**5. We note that Figure 1 in your submission contain [map/satellite] images which may be copyrighted. All PLOS content is published under the Creative Commons Attribution License (CC BY 4.0), which means that the manuscript, images, and Supporting Information files will be freely available online, and any third party is permitted to access, download, copy, distribute, and use these materials in any way, even commercially, with proper attribution. For these reasons, we cannot publish previously copyrighted maps or satellite images created using proprietary data, such as Google software (Google Maps, Street View, and Earth). For more information, see our copyright guidelines:** [**http://journals.plos.org/plosone/s/licenses-and-copyright**](http://journals.plos.org/plosone/s/licenses-and-copyright)**.**

**Response:**

Thank you so much. Figure 1 was drawn by the authors, therefore, the authors own the copyright of Figure 1. The authors grant permission for the open-access journal PLOS ONE to publish Figure 1 under the Creative Commons Attribution License (CCAL) CC BY 4.0.

**Reviewer #1:**

This study examines how the introduction of long-term care insurance in China has affected the amount of family care provided to older adults using a difference-in-differences analysis approach, taking into account the heterogeneity of the subjects. The results show that the introduction of long-term care insurance leads to a decrease in family care. They also show that the impact of insurance varies depending on the attributes of the target population. The authors demonstrate the effectiveness of long-term care insurance.

This paper provides important insights into how to support family caregiving for older adults in an aging society. The hypothesis is clearly presented, the analysis is well designed, and the results are interesting. However, as interesting as the study is, it is very disappointing that the authors disregard the journal's author guidelines. In addition, the validity of the data used is unclear and there seems to be insufficient consideration of limitations. I hope you will take the following points into consideration.

**Major comments**

**1. The author guidelines contribute to readability, so the authors should be divided accordingly into Introduction, Materials and Methods, Results, Discussion, and Conclusions. I understand that Results and Discussion are sometimes written together in economics and policy studies, but at least in 4.4.2, 4.5, and Table 8, analyses not described in Methods should not be started in the Results and Discussion sections. The Methods section should include a description of the analyses for all robustness checks and heterogeneity analyses, as well as the analyses that accompany them (e.g., balance test). The Vancouver method should also be used for references in accordance with the guidelines.**

**Response:**

Thank you for your suggestions. We have revised the manuscript as follows:

Firstly, according to the author's guidelines, we have revised the article structure into following 6 sections: Introduction, Policy background and research hypothesis, Materials and methods, Results, Discussion, Conclusions and policy implications. Based on the original manuscript, we also added a section named "Policy background and research hypotheses" to introduce more details of the pilot long-term care insurance policies in China. We hope that’ll help provide more background information for readers.

Secondly, we have revised the structure of the methods, results and discussion sections. In the "methods" section, we have presented an overview of all the primary research methods conducted in this study. We have consolidated all the empirical analysis in the "results" section. Through these modifications, we have ensured that the "methods," "discussion," and "results" sections are more in line with the basic requirements of the journal.

**2. 2.1 Policy background: Please consider adding a description of the system (e.g., delivery system, support system, etc.) for medical care and long-term care for older people in China before the intervention begins. My concern is that when financial support is provided through long-term care insurance, residents will not be able to use them if the local service delivery system is not adequate. Also, the medical care delivery system could complement long-term care services.**

**Response:**

Thank you so much for this valuable comments. We have rewritten the entire "policy background" section. Please see from page 3 to page 5. In the revised version, we introduced the system in two sub-sections: the first primarily analyzes the differences between China's medical care and long-term care insurance, while the second provides a brief overview of the development and basic situation of long-term care insurance. In China, Long-term care insurance is composed of separately established healthcare institutions that introduce market entities such as nursing homes and medical institutions to provide services. Based on our research in various regions, it is observed that older adults who qualify for long-term care insurance can generally receive comprehensive and qualified nursing services. In addition, medical care covers expenses related to the prevention and treatment of chronic diseases and illnesses in older adults, while long-term care insurance primarily provides service support for disabled older adults.

**3. 2.1 Policy background: Please consider specifying the differences between medical care and long-term care in the Chinese system. In particular, it would be helpful to explain the differences in financing, payment, service delivery systems, and service content.**

**Response:**

Thank you for your suggestion. We have discussed the differences between China’s medical insurance and long-term care insurance in the policy background section on page 3 and 4.

**4. Figure 1: It would be easier to understand if you could visualize which regions are starting to introduce the system and from which survey year. I am not familiar with Chinese place names, so I have difficulty understanding the text and figures; I suggest numbering the pilot areas and indicating the place names with notes. Also, please let me confirm what is the difference between the pilots by the local government and the officially announced 15 pilots.**

**Response:**

Thank you for your suggestions. I have made the following revisions to the manuscript:

Firstly, Figure 1 presents the basic situation of the pilot program for the long-term care insurance in China. The subsequent research in the manuscript is based on the treatment groups annotated in the CHARLS data from the regions depicted in Figure 1. We totally agree with you that adding the specific policy implementation year for each pilot city will make more sense for the readers while reading this paper. We have added detailed policy implementation years for all the pilot cities in the "Note" section of Figure 1.

Secondly, the officially announced 15 pilot cities are national-level pilot cities that have been determined by the central government through the issued documents. They are part of a formal national policy promoted by the central government. On the other hand, local government pilots are initiated by the local governments themselves and are local policies undertaken to address the long-term care needs of older adults. The general pattern of institutional development in China is to first select representative regions for regional pilots, gradually expand the scope of the pilot program, and eventually implement it nationwide.

We really appreciated the suggestion of numbering the pilot cities to help readers distinguish different cities. But after careful consideration, we did not number the pilot cities in this revised version, instead, we added the specific policy implementation year for each city to help readers distinguish the pilot cities.

**5. 2.2 Literature Review: Prior studies have been properly reviewed, but it would help the reader's understanding by specifying which country the study is from.**

**Response:**

Thank you for your suggestion. It is indeed important to acknowledge that different scholars’ perspectives are based on different countries and regions. We have made efforts to indicate the countries associated with different viewpoints as much as possible, so that readers can have a clearer understanding of this.

**6. 3.1 Data: CHARLS data is insufficiently detailed. At the least, the survey's subject and target sampling methods, survey methodology, geographic areas included, response rates, and dropout rates are important for bias and interpretation of the results. Please consider providing explanations in the text and supporting materials or citing articles that describe them.**

**Response:**

Thank you for your comments. Our description of the data source is primarily based on the official website of CHARLS, which can be found at http://charls.pku.edu.cn/gy/gyxm.htm. In response to your suggestion, we have added additional detailed information in the data section (page 8) to provide more detailed information about the data. This ensures that our data contains sufficient details.

The followings are the data details we added in this revision:

(1) Sponsor of the data Project: Peking University National Development Research Institute, Peking University China Social Science Survey Center, and Peking University Youth League Committee.

(2) Start time and frequency of follow-up surveys: The survey started in 2011, and three follow-up surveys were conducted in 2013, 2015, and 2018.

(3) Coverage: The survey covers 28 provinces in China and includes nearly 20,000 respondents from 450 communities nationwide (Excluding Hong Kong, Macau and Taiwan Province).

(4) Main methodology: The questionnaire design drew on international experiences, including the Health and Retirement Study (HRS) in the United States, the English Longitudinal Study of Aging (ELSA), and the Survey of Health, Aging, and Retirement in Europe (SHARE), among others. The project employed a multi-stage sampling approach, with the use of probability proportional to size (PPS) sampling at both the county/district and village levels. CHARLS pioneered the use of electronic mapping software (CHARLS-GIS) technology to create village-level sampling frames using a mapping method.

(5) We also added the detailed description of the two reasons, process, and results of using CHARLS in this study.

Unfortunately, CHARLS did not disclose response rates, therefore we were not able to talk about it in the article. But we made sure that we include detailed sample and variable information in the descriptive statistics section. Thank you for your understanding and support.

**7. please specify how many subjects were excluded in each of the two stages of the selection process.**

**Response:**

Thank you for your suggestion. I have added the number of lost samples during the two stages of processing on page 8.

**8. Were there any missing data? If so, how were they addressed?**

**Response:**

Thank you for your comments. Yes, there were missing data in the variables "Upward" economic support and "Downward" economic support. Since these two variables are continuous, we used the interpolation method to handle missing values by predicting them based on neighboring observations. Taking your advice into consideration, we have added supplementary explanations in the note section of Table (page 13).

**9. 3.3.1: Individuals are nested in cities. Therefore, I am concerned that each observation is not independent over time and that there is a city-level autocorrelation. For example, have you considered using city cluster standard errors or using city random effects?**

**Response:**

Thank you for your comment. We greatly agree with your point of view. In the basic regression, we only controlled for time and individual fixed effects, without considering city random effects. Therefore, we have made modifications in the "robustness test" section. In this section, we have added city and province fixed effects, as well as some interaction terms. We hope that this approach will help address the issue.

**10. 3.3.2: Did the authors use "nearest neighbor matching within caliper"? Please provide more clarification on the matching method.**

**Response:**

Thank you for your suggestion. The standalone PSM method provides multiple matching methods, and the PSM-DID model typically uses default kernel matching for estimation. In accordance with your suggestion, we have made modifications to the corresponding section and explained the method we used.

**11. 3.3: Did they use linear regression for all analyses?**

**Response:**

Thank you for your comment. The dependent variable in this study is a continuous variable, and the data used is panel data. Therefore, the main research method employed is the linear regression method (“xtreg”). In the mechanism analysis, intergenerational economic support is also a continuous variable. Therefore, it is mostly analyzed using the linear regression method, unless otherwise specified.

**12. Table 1: Considering the DID, it would be easier to understand the changes if you show the mean values before and after the intervention for each group, respectively.**

**Response:**

Thank you for your comment. We have made supplementary modifications to Table 1, adding the mean and SD before and after intervention.

**13. Figure 2: Does "current" on the horizontal axis indicate the time of intervention? How is it taken into account in this analysis if there is only one time point before the intervention or one time point after the intervention?**

**Response:**

Thank you for your comment. We have made modifications based on the data implementation period, time, and your feedback. The term "current" represents the relative period of the policy pilot, indicating the current intervention.

**14. 4.4.1: Was the overlap of the propensity score values sufficient?**

**Response:**

Thank you for your comment. We have reported the basic information of the relevant variables before and after matching in Table 3, and the balance test results have passed. We have also provided supplementary explanations in Table 3 regarding the changes and losses in the samples before and after matching to address the issue you raised. The content is as follows:

“In addition, after performing propensity score matching, there were 495 samples in the control group that were not within the common range of values. However, all samples in the treatment group were within the common range. As a result, only a small number of samples (approximately 2.6%) were lost during the propensity score matching process.”

**15. Line 608: What do the authors consider the validity of the common shocks assumption for DID in this study?**

**Response:**

Thank you for your comment. I’m not quite clear on the reason behind your question regarding “Line 608,” so I’m not entirely sure what you mean. However, my understanding is that the assumptions of the Difference-in-Differences (DID) model primarily include three points:

Assumption 1: Linearity assumption: The latent variable, treatment variable, and time variable satisfy the linear condition.

Assumption 2: Individual treatment stability assumption: Policy intervention only affects the treatment group and does not have any interactive effects on the control group.

Assumption3: Parallel trends assumption: Before policy intervention, the treatment group and control group should have similar trends in outcome effects.

**16. Policy Implications: We should limit ourselves to suggestions that can be made based on the results analyzed in this study. Some of the suggestions seem to be overstated.**

**Response:**

Thank you for your comment. We have made modifications to the policy recommendations to ensure that each recommendation is based on the research findings.

**Minor comments**

**1. 3.3.3: Equations 7 and 8 are missing.**

**Response:**

Thank you for bringing this issue to our attention. We apologize for the mistake in numbering, and we have made the necessary corrections in the manuscript.

**2. Table 4: seems to contain Chinese characters.**

**Response:**

Thank you for your reminder. We have made the necessary corrections in the corresponding section of the manuscript.

**3. Line 594: Table 9 is missing.**

**Response:**

Thank you for your reminders. The manuscript has undergone multiple revisions, leading to some numbering errors. We have made the necessary corrections in the corresponding sections of the manuscript.

**Reviewer #2:**

**1. There is much room for modification in the language, typesetting and grammatical methods of the article.**

**Response:**

Thank you for your feedback. We sought the assistance of professional English experts and have made the necessary modifications to the grammar and other issues in the manuscript to ensure it meets the journal's requirements.

**2. Please strengthen introduction section to highlight the value of this study. Besides, each paragraph in the introduction seems to be unrelated to the previous paragraph.**

**Response:**

Thank you for your comments. This article provides a detailed introduction to the background and marginal contribution of our research. The first paragraph discusses the severe situation of population aging and disabled population. The second paragraph highlights the heavy burden of family caregiving in this context. The third paragraph emphasizes the importance of long-term care systems. The fourth paragraph describes the contribution of our study.

**3. There is an expression mistake, not '家庭照料' but 'family care'.**

**Response:**

Thank you for your reminder. We have made the necessary corrections in the corresponding section of the manuscript.

**4. Regarding sample selection, to enhance the reliability of research conclusions, the author needs to accurately match pilot samples.**

**Response:**

Thank you for your comment. We first conducted a balance test on the relevant variables in the propensity score matching process and provided a detailed description of the matching process and sample characteristics. Our results meet the basic requirements of the matching assumption. Thank you for your support and understanding.
