# Supplementary material for: The impact of long-term care insurance on family care for older adults: The mediating role of intergenerational financial support
Source: PLoS One. 2024 May 23;19(5):e0299974. doi: 10.1371/journal.pone.0299974 (PMC11115205; doi:10.1371/journal.pone.0299974)
Supplement: Attachment — Submitted filename: Response to Minor Revision.docx [file pone.0299974.s002.docx]

**Response to Minor Revision**

Dear Editor and Reviewers,

We would like to express our gratitude for your professional suggestions and constructive comments, which are essential for improving the quality of our research paper. We also greatly appreciate your recognition and acceptance of our paper. Based on some suggestions for minor revisions, we have made the following modifications to our paper:

**Reviewer #1:**

I would like to express my gratitude to the authors for sincerely addressing the comments and making the necessary amendments. These changes have improved the quality of the manuscript. However, it seems that the description in the Methods section remains insufficient. According to the STROBE guideline, the methods for all sensitivity analyses must also be included in the Methods section. Please consider adding details about Robustness tests, Heterogeneity analysis, and Further analysis in the Methods section. It is better to refrain from presenting additional analytical methods in the results section.

Another concern is the lack of published response rates for the CHARLS data. This omission could potentially introduce a significant bias in the results, so I recommend acknowledging this as a limitation.

Overall, nice work. I hope this research contributes to the development of China's long-term care insurance system.

**Response：**

Thank you for your professional feedback and recognition of our manuscript. Your meticulous and professional attitude is commendable. Taking your suggestions into account, we have made the following modifications to the paper:

Firstly, we have reorganized the methodology section based on your suggestions. We have rearranged and elaborated on the methods for different stages in the order of empirical analysis in our paper. We have removed statements describing the methodology in the "Results" section to align it as closely as possible with your requirements.

Secondly, we have added a research limitation regarding the disclosure of response rates in the CHARLS dataset. In fact, based on the data processing results, the response rates for various questions in the CHARLS dataset exceed 90%, ensuring the reliability of the findings in this study.

Other modifications:

We made some slight revisions to the title to ensure a more comprehensive expression of the research content. Additionally, we have also made some formatting adjustments based on previously published papers in this journal.

Finally, we would like to express our heartfelt gratitude for your thorough work. We wish you good health and a happy life in the new year.
